# Development, validation, and implementation of a Short Breast Health Perception Questionnaire

**DOI:** 10.1186/s12889-022-13501-5

**Published:** 2022-05-27

**Authors:** Sadaf Alipour, Hadi Rashidi, Khadije Maajani, Marzieh Orouji, Yas Eskandari

**Affiliations:** 1 Breast Disease Research Center (BDRC), Cancer Institute, Tehran University of Medical Sciences, Tehran, Iran; 2 Department of Surgery, Arash Women’s Hospital, Tehran University of Medical Sciences, Tehran, Iran; 3 Department of Epidemiology and Biostatistics, School of Public Health, Tehran University of Medical Sciences, Tehran, Iran; 4 Department of Nursing, Arash Women’s Hospital, Tehran University of Medical Sciences, Tehran, Iran; 5 Faculty of Psychology and Education, University of Tehran, Jalal Al-Ahmad St, Tehran, Iran

**Keywords:** Beast Health, Questionnaire, Validity, Reliability, Content

## Abstract

**Background:**

Health status and perception can be assessed by general or disease-specific questionnaires, and disease specific questionnaires are more specific than general questionnaires. Considering the importance of breast health perception (BHP) in women’s lives and the lack of any pertinent questionnaires, we performed this study to develop a valid and reliable short BHP questionnaire (BHPQ); and then used it to assess the participants’ BHP.

**Methods:**

We first designed and developed the instrument and then measured its inter-rater agreement (IRA), content validity including content validity index (I-CVI) and scale content validity index (S-CVI), and reliability (through internal consistency and test–retest). We then evaluated the BHP of eligible women with normal breasts and benign breast disorders who attended our breast clinic.

**Results:**

The IRA index (78.6%) showed the optimal relevance and clarity of the questionnaire. The content validity was acceptable; with S-CVIs of 87.35 and 84.42 for clarity and relevance, respectively. The internal reliability was high (Cronbach’s alpha = 0.93). Three questions were eliminated for internal consistency (intraclass correlation coefficient < 0.7) but the rest of the questions showed good and excellent reliability. In the next step, BHP in the 350 eligible participants showed an overall score of 43.89 ± 9.09.

**Conclusion:**

This study introduces a valid and reliable 11-item BHPQ. We propose its use in various circumstances throughout breast cancer screening, diagnosis, and treatment; and in the assessment of BHP in various physiologic and reproductive situations.

## Background

Cancer is the second leading cause of death worldwide, and breast cancer is the most common cause of cancer-related mortality [1]. The breast is a distinctive feature of women and an emotional symbol of femininity and motherhood, and any threat to the breast can be devastating to the feminine identity of a woman [2]. A diagnosis of breast cancer can be destructive and induces various negative reactions in most women [3]. In addition, the loss of a breast can result in low self-esteem, false self-perception, social isolation, and communication problems with others [4, 5]; while the psychological health of the operated woman can diminish, leading to poor health outcomes [6, 7]. Thinking about the possibility of developing breast cancer itself can cause intense mental stress, which can evoke different emotional problems such as anxiety, distress, and depression [2, 8].

Breast cancer screening can facilitate early detection of malignancy, improve the patient’s quality of life and reduce cancer-related mortality [9]. However, being called for further investigations after primary screening is a stressful experience for many women [10], and false negatives, false positives, and overdiagnosis can affect the patients’ decision to participate in the screening program [11]. In addition, psychological reactions such as anxiousness and depression are common during the detection, diagnosis, and treatment of cancer [12]. These procedures and events cause different levels of stress in women, and they might also affect the patient’s perception regarding her breast health. Regardless of the real health status, health perception is important enough to affect a person’s life satisfaction -as defined and measured by Emmons and Diener- and quality of life (QOL) [13, 14]. Interestingly, it has been shown that when patients rate their health perception, they do not merely envisage their physical status, but also other elements such as general well-being. When people evaluate and rank their health status, they point to data that can even predict the probable incidence of chronic diseases or recovery from the disease, their functional deterioration, or their future use of health services [15]. Therefore, professionals like to assess a person’s health perception alongside their physical and emotional health in various conditions. This assessment takes place via questionnaires that have been designed for this purpose [15]. Overall, general health feelings are important aspects of medical care and can be assessed by general or disease-specific questionnaires. The general health questionnaire, including its frequently used short version consisting of 12 questions, has been translated and validated in multiple languages and countries and is the best-known questionnaire in this regard [16]. However, site or disease-specific questionnaires are more sensitive for detecting and quantifying small issues [17]. Numerous valuable specialized questionnaires focusing on an important body organ or a specific disease that measure a person’s health perception, health status, or quality of life in relation to that disease have been developed and validated. In 2014, Oldridge et al. validated a questionnaire that was specifically aimed at patients with ischemic heart disease. It comprised 14 items including physical and emotional subscales and a global score, and the Cronbach’s alpha index for consistency was > 0.8 [18]. Another study conducted in the Netherlands for validation of QOL after pulmonary embolism reported an adequate internal consistency with a Cronbach’s alpha of 0.62–0.94 and a reliability of 0.78–0.94 [17]. A few other examples among many include peripheral artery [19], cystic fibrosis [20], leiomyomata [21], sleep apnea [22], and onychomycosis [23] questionnaires.

However, despite the importance of the breast in a woman’s life, a questionnaire that targets the perception of women toward their breast health (BH) is not available. Although questionnaires for assessing breast cancer fear (the Champion Breast Cancer Fear Scale) [24] and patient-stated outcome after breast reduction, augmentation, and reconstruction have been validated (the Breast-Q) [25]; no questionnaires for evaluation of BH perception (BHP) have been introduced until now. While the threat of cancer and its burden can be destructive and result in depression and anxiety in women, many other issues like harboring a premalignant lesion in the breast, benign breast disorders, various types of breast surgery, mild and moderate breast symptoms like mastalgia or benign nipple discharge, physiologic reproductive conditions such as pregnancy, lactation, or menstrual fluctuations of the breast, and even simply undergoing cancer screening can impact the BHP. Therefore, developing a questionnaire with acceptable validity and reliability to evaluate women’s self-perception about their BH can be very helpful. The purpose of this breast health perception questionnaire (BHPQ) would be to detect how women perceive their breast health when facing different situations such as undergoing any kind of investigation for detection of a breast lesion, the diagnosis of a benign breast disease, encountering changes in the breasts due to pregnancy or lactation; becoming aware of the disease of an acquaintance, or taking part in a study about breast medical conditions, or any other event related to the breasts.

We perceived the need for a BHPQ while executing a breast cancer screening project during preconception care in women. At that time, we realized that the clinical and imaging examinations worried some women, but we needed to know whether our activities had any impact on the women’s BHP. Similarly, when we were carrying out a study about two different methods of educating women about breast cancer [26], we saw that the instructions we provided about breast cancer made some women feel uncomfortable about their breasts. We then looked for a tool that could measure the BHP of women before and after the teaching interventions to compare the two methods and see whether any of them could have a better impact or inflict less damage to the participants’ BHP. These and many other similar evaluations could only be done by using a reliable, validated, specifically designed questionnaire. Unfortunately, such a tool does not exist. Therefore, we carried out the present study to develop a valid and reliable short questionnaire for measuring women’s perception about their BH status. We then used the product to assess BHP in women with normal breast and with benign breast disorders who attended our breast clinic.

## Methods and materials

This study has been approved by the Research Deputy of Tehran University of Medical Sciences, approval code 99–1-259–48,164. Also, the study has been approved by the Ethics Committee of Tehran University of Medical Sciences, ethics code: IR.TUMS.IKHC.REC.1399.112. All the participants consented to take part in the study by written informed consent. All methods were carried out in accordance with relevant guidelines and regulations of the ethics committee of the University of Medical Sciences and the declaration of Helsinki.

### Questionnaire design

To design a BHPQ, we first performed an extensive literature review. Then, several discussion panels with 7 general surgeons, breast surgeons, and gynecologists were held. During these meetings, the content that should be included in the questionnaire, as well as the content that should not be included, were discussed (content validity assurance). Fourteen multiple-choice questions were developed and the answers were set based on the Likert scale, including always (almost always), usually, sometimes, rarely, and never.

### Validity assessment

#### Face validity

Face validity is used to assess whether a questionnaire is appropriate for the measurement of what is expected to be measured in general [27]. To do this, the approved questions were given to 12 experts (other than those who had taken part in the development of the questions) to evaluate them regarding their form, comprehensibility, sequence, and fluency. The experts were chosen according to the recommendation of the research directory reviewers of the university and included two breast surgeons, two surgical oncologists, one general surgeon expert in breast diseases, two gynecologists, two radiation oncologists, one psychologist, one social medicine expert, and one breast care nurse.

#### Content validity

Before handling the questionnaires to the experts, several patients were asked to evaluate the paper to consider whether they believed it could measure a woman’s perception about her breast health; and give us their oral opinion or approval. They did not rate the questionnaire officially in this regard, as this part of the work had not been planned in the project.

Relevance is the ability of a selected question to reflect the characteristics of the intended content, while clarity addresses whether a selected question is appropriate in terms of writing and its concept. Because clarity and relevance have different definitions, we asked a panel of experts to rate every item in terms of relevance and clarity separately according to a four-point Likert scale. Thus to conduct content validity [28], the designed questionnaire was given to these 12 health professionals and 5 knowledgeable participants as an expert panel, to score the indices of content validity (relevancy and clarity) for each item in the questionnaire (I-CVI) according to the four-point Likert scale.

##### Item Content Validity Index (I-CVI)

This index rates the relevance and the clarity of each item in the questionnaire [27, 28]. To obtain each index, the number of experts judging each item as relevant or clear (rated as “quite appropriate” or “appropriate”) was divided by the total number of experts. Returned values range from zero to one: As a rule, when the item content validity index (I-CVI) is more than 0.79 the item is relevant, when it is between 0.70 and 0.79 it needs revision, and if the value is below 0.70, the item is eliminated [28].

##### Scale Content Validity Index (S-CVI)

There are two methods used to assess the relevance and the clarity of the overall scale: total agreement and mean approach. In both approaches, the “quite appropriate” and “appropriate” answers, as well as the “quite inappropriate” and “inappropriate” are merged; and two main options “appropriate” and “inappropriate” are considered for each question.

For the total agreement approach, the number of questions rated as appropriate is divided by the number of questions. In the mean approach, the total index of content validity (relevancy or clarity) of every item in the questionnaire is divided by the total number of questions.

In different references, the minimum acceptable scale content validity index (S-CVI) for a new instrument is considered to be 80% [28].

### Interrater agreement

The interrater agreement (IRA) is used to examine the observed agreement between experts participating in a study in terms of relevance and clarity of questions [28]. The assessment of IRA is performed in two different ways, conservative and less conservative. For the conservative approach, the number of items that all experts rated as “quite appropriate” or “appropriate” was divided by the total number of items. For the less-conservative approach, the number of items that the majority of experts (80%) rated as “quite appropriate” or “appropriate” was divided by the total number of items. The acceptable level of agreement was presented as 70%-80% [29].

### Reliability

Reliability is the degree to which the research method produces stable and consistent results under the same method and circumstance over time [30]. We used Cronbach’s alpha to determine the internal consistency and the test–retest method for reliability. Since in this study, all items (questions) were related to one area, then only one Cronbach’s alpha index was estimated for the instrument. In internal consistency evaluation, the desired value for Cronbach’s alpha is 0.7 or higher. To evaluate the reliability of the instrument, questions were given to a group of 36 people at two different times with 2 to 3 week intervals under the same circumstance, and the obtained scores were used to measure the reliability by using the intraclass correlation coefficient (ICC). Reliability above 0.7 is desirable [31, 32].

### Study design

After designing a valid and reliable questionnaire, a descriptive-analytical and cross-sectional study was carried out on women who attended the Breast Clinic of Arash Women’s Hospital from August 1st to October 31st, 2020. The inclusion criteria of the study were: age 18 years or above, willingness to participate, no suspicious breast lesions on clinical breast exam, no suspicious breast lesions on breast ultrasound (when needed), and the absence of any suspicious lesions on mammography during the past year in women over 40 years of age. Exclusion criteria consisted of a history of breast cancer, a diagnosis of a benign breast lesion except for fibrocystic changes or small (less than 1 cm) fibroadenomas, a history of cosmetic breast surgery, a new change in the breast examination or in a recent imaging examination, a history of psychological disease, or the use of psychotropic medications. After getting written informed consent from the eligible women, 350 women were entered into the study. Sampling was done in a full-census manner and all eligible participants were selected to fill out the BHPQ. Each question (item) included five-choice options; always (almost always), usually, sometimes, rarely, and never; which were given one to five scores, respectively. The overall calculated raw score for each participant could be between 14 and 70 in the designed questionnaire.

## Results

### Content validity

I-CVI and S-CVI were used to assess the content validity. I-CVI values for relevance and clarity were between 58.8 and 100, and S-CVI was 87.35 and 84.42, respectively. The calculated IRA for the BHPQ was 78.6. (Table1).

**Table 1:** Items content validity index (I-CVI), scale content validity scale (S-CVI) and IRA for clarity and relevancy

Question	Clarity				Relevancy			
	S-CVI (Mean approach)	IRA	I-CVI	Number of agreements among 17 observed	S-CVI (Mean approach)	IRA	I-CVI	Number of agreements among 17 observed
Q.1	87.35	78.6	82.3%	14	84.42	78.6	100	17
Q.2			82.3%	14			82.3	14
Q.3			64.7%	11			58.8	10
Q.4			70.5%	12			70.5	12
Q.5			94.1%	16			94.1	16
Q.6			94.1%	16			94.1	16
Q.7			100%	17			94.1	16
Q.8			94.1%	16			82.3	14
Q.9			100%	17			94.1	16
Q.10			76.5%	13			82.3	14
Q.11			100%	17			88.2	15
Q.12			88.2%	15			82.3	14
Q.13			88.2%	15			88.3	15
Q.14			88.2%	15			70.5	12

*I-CVI* Item Content Validity Index, *S-CVI* Scale Content Validity Index (with mean approach), *IRA* Inter Rater Agreement

### Reliability

To assess the internal consistency based on the Likert scale (Q1 to Q14), we used Cronbach’s alpha. According to the results, the internal consistency of the BHPQ was excellent (Cronbach’s alpha = 0.93). The reliability of the instrument was measured by the ICC by comparing the total score of the questionnaire filled by people at two different times (with a 2 to 3-week interval).

The calculated ICC index for qualitative variables showed that the internal consistency of most of the questions was acceptable; questions with an ICC < 0.7 were removed from the questionnaire (Table 2).

**Table 2:** Test–retest reliability

Question	Intraclass correlation Coefficient (ICC)
Q.1	0.81
Q.2	0.89
Q.3	0.73
Q.4	0.71
Q.5^a^	0.50
Q.6	0.71
Q.7	0.80
Q.8	0.74
Q.9^a^	0.34
Q.10	0.81
Q.11	0.76
Q.12	0.71
Q.13	0.75
Q.14^a^	0.43

^a^Excluded questions

### Final questionnaire

The minimum and maximum overall scores of the questionnaire were considered as 14 and 70, respectively, at the time of designing the questionnaire. After the removal of the questions with a low ICC during the reliability assessment, the overall score was 11–55. The specific main topics asked in the questionnaire evaluated the subject’s feelings about the current or future presence of a dangerous disease in her breast; her anxiety and worries about getting her family into trouble, the worry of her present or future breast problems disrupting her daily life or interfering with her sexual relationship; the urge for repeated medical check-ups to check the breast health status; and matters such as obsessive breast self-exam and frequent searching or inquiring for information about breast diseases or new ways to diagnose breast lesions.

The questionnaire is in Farsi but a translation of the questions as well as the answers options (on a five-point Likert scale) and the scoring are demonstrated in Table 3.

**Table 3:** The English translation of the final breast health perception questionnaire

	**Question (translated from Farsi)**^a^	**Answers**
1	I feel I have a dangerous disease in my breast	*On a Likert scale:* Always = 1 point Very often = 2 points Sometimes = 3 points Rarely = 4 points Never = 5 points
2	I feel I will get a dangerous disease in my breast in the future	
3	I feel that I am causing trouble for my family due to my breast conditions	
4	I feel I have a disorder in my breast that will cause troubles for my family in the future	
5	I feel I have a problem in my breasts and this thought makes me anxious	
6	I feel I have a problem in my breasts and this thought has disturbed my daily life	
7	I feel I have a problem in my breasts and this thought disrupts my sexual activities	
8	I need to obsessively examine my breasts to stay calm	
9	I need to go for breast checkups sooner than my doctor has recommended for my peace of mind	
10	I constantly search for and inquire about new methods for detection of breast disorders	
11	I am constantly on the search for new information about breast diseases	

^a^The validation of the questionnaire was done on the Farsi version for Iranian patients

### Participant’s evaluation

A total of 350 women were included in the study. The mean age of the participants was 42.7 ± 10.29 years. The youngest participant was 18 and the oldest was 83 years old.

The overall mean score of BHP among the 350 women was 43.89 ± 9.09.

## Discussion

This study aimed to develop a new questionnaire for measuring BHP in women, assess the validity and reliability of the instrument, and evaluate the status of BHP in a group of women who did not have breast cancer and were not at immediate risk for the disease according to imaging and physical examination. Results of the study provided evidence that the designed tool yielded a reliable and valid 11-item BHPQ for evaluating BHP. The process started with designing and developing the instrument, followed by evaluation of IRA, content validity including I-CVI and S-CVI, and finally reliability (through internal consistency and test–retest).

Based on the results, the IRA index obtained for the instrument (78.6%) indicated optimal relevance and clarity of the questionnaire. Also, the content validity of the final BHPQ was acceptable: the S-CVI, which is one the most important indices in designing an instrument [28], was acceptable (87.35 and 84.42 for clarity and relevance, respectively). Reliability is a term used to describe the consistency of a measure. Essentially, if findings can be replicated consistently, they are considered to be reliable [33]. In our study, the internal reliability of the BHPQ was found to be high with a Cronbach’s alpha of 0.93, which shows the excellent consistency of the questionnaire. For external consistency, the three questions that showed an ICC less than 0.7 were removed from the questionnaire, while the rest showed good and excellent reliability. In the end, a valid reliable 11-item BHPQ was developed.

Our study was not meant to define a cut-off point for breast health perception. We only intended to provide a score which could be used as a comparative measurement. A higher score meant a better condition, and a lower score indicated a poorer condition. Also, the changes before or after an intervention would show the positive or negative effect of the intervention on the BHP.

In the second part of the study, measuring BHP in the eligible women who attended the Breast Clinic of Arash Women’s Hospital showed an overall score of 43.89 ± 9.09. According to the findings, among different variables which could affect BHP, age was the only one that showed a significant relationship with BHP; older women showed a higher BHP score. It might be assumed that this is because understanding and correctly answering questions on a survey are more difficult for older people and the results are biased. However, it has been shown that short questionnaires usually do not induce such a bias, contrary to lengthy ones [34]. Also, it has been mentioned that life satisfaction (defined as “desire to change life or satisfaction with current/past/future life or others' views of one's life”) usually does not decrease with increasing age [35], and even living with a disease beyond a certain time leads to increased levels of well-being [36]. This finding in our study might be attributed to the fact that older women had lived a longer time without any important breast lesion, and were somehow reassured about their BHP.

Our study showed that the history of breast surgery for benign lesions did not affect BHP. A study conducted by Klassen et al. revealed that cosmetic and reconstructive breast surgery affect the psychological well-being of women [37], and higher satisfaction and psychological well-being were detected after breast reconstruction in the study of Ng et al. [38]. However, these types of surgeries aim to resolve a defect or compensate for a subject of dissatisfaction of the patients and expectedly yield positive feelings. Nevertheless, the BHP of these patients has not been evaluated in these or other studies; our questionnaire can be used for this purpose in the future.

Having a family history of breast cancer was evaluated as a potential feature that could affect BHP, but according to the results, there was no significant difference between women with a positive or negative family history. This finding is consistent with a previous study conducted by Al-Naggar et al., which reported that a family history of cancer does not influence the QOL [39]. However, Abu-Helaleh et al. reported positive family history of breast cancer as an important predictor of low QOL [40].

Other factors, which were considered and evaluated as a potential predictor of BHP, were menopause, lactation, parity, and miscarriage; none of them showed any significant relationship with BHP.

Our study had some limitations. We did not ask patients to assess the validity of the questionnaire and only asked for their oral approval. We did not differentiate women with healthy breasts from those with mild benign disorders (fibrocystic changes and small fibroadenomas). Also, we did not include women who had undergone previous cosmetic breast surgery, and only evaluated surgery for benign lesions. In addition, we did not evaluate the BHP status of patients with previous breast cancer. The BHPQ is now in Farsi, and the English translation and validation should be performed as the next step to make the BHPQ usable for a wide population.

## Conclusion

This study introduces an 11-item BHPQ that can be used in various situations for the assessment of BHP. We propose its evaluation in breast cancer survivors at different stages of diagnosis, treatment, survivorship, and follow-up; and in women at high risk for breast cancer. Also, we suggest using this questionnaire in various conditions throughout breast cancer screening, and in projects which might affect the self-perception of women about the health status of their breasts.

## Data Availability

The data that support the findings of this study are available from the first author of the manuscript by correspondence to sadafalipour@yahoo.com.

## Abbreviations

BH: Breast health
BHP: Breast health perception
BHPQ: Breast health perception questionnaire
I-CVI: Content validity index
S-CVI: Scale content validity index
ICC: Intraclass correlation coefficient
